# Dimensionality reduction for images of IoT using machine learning

**DOI:** 10.1038/s41598-024-57385-4

**Published:** 2024-03-26

**Authors:** Ibrahim Ali, Khaled Wassif, Hanaa Bayomi

**Affiliations:** Computer Science Department, Faculty of Computers and Artificial Intelligence, Cairo University, Giza, Egypt

**Keywords:** Edge computing, Deep learning, IoT, Autoencoder, Computer science, Information technology, Software

## Abstract

Sensors, wearables, mobile devices, and other Internet of Things (IoT) devices are becoming increasingly integrated into all aspects of our lives. They are capable of gathering enormous amounts of data, such as image data, which can then be sent to the cloud for processing. However, this results in an increase in network traffic and latency. To overcome these difficulties, edge computing has been proposed as a paradigm for computing that brings processing closer to the location where data is produced. This paper explores the merging of cloud and edge computing for IoT and investigates approaches using machine learning for dimensionality reduction of images on the edge, employing the autoencoder deep learning-based approach and principal component analysis (PCA). The encoded data is then sent to the cloud server, where it is used directly for any machine learning task without significantly impacting the accuracy of the data processed in the cloud. The proposed approach has been evaluated on an object detection task using a set of 4000 images randomly chosen from three datasets: COCO, human detection, and HDA datasets. Results show that a 77% reduction in data did not have a significant impact on the object detection task’s accuracy.

## Introduction

In recent years, the explosion of sensors, wearables, mobiles, and other Internet of Things (IoT) devices has been changing how we live and work. The applications of IoT services have started pervading all industrial sectors, from smart homes and cities to education, healthcare, transportation, supply chain management, and logistics. There are many forecasts for the huge growth of the IoT. Analysts predict that there will be 41.6 billion connected IoT devices^1^, and the global economic impact of the IoT will be between USD 2.7 trillion and 6.2 trillion by 2025^2^.

Image data from IoT sensor devices are exchanged over the network for storage, processing, or control. To realize the benefits of smart IoT systems and extract value from collected images, data analytics is essential in the cloud by transferring this data to the cloud for storage and processing. For example, images from a smart transportation system are transferred to a far-off data center for storage and processing. Attempting to transfer all those images to the cloud for processing will increase latencies and put a strain on communication networks. Those connected devices are limited in the analytics they can perform because of limited computation power, storage capacity, and other factors.

Edge computing is a distributed computing paradigm that brings processing and data storage closer to the sources of data. This is expected to improve response times and save bandwidth^3^. It is an architecture rather than a specific technology. The edge server acts as a connection between a private network in an organization and the cloud. It can be used for processing offloading and can act as an intermediary between the cloud and IoT devices by performing a reduction on data and sending the reduced data to the cloud for further processing.

Deep learning^4^ is a subclass of machine learning (ML) that plays a vital role in creating a smarter IoT. It has shown remarkable results in various fields, including dimensionality reduction and image recognition. The combination of deep learning and dimensionality reduction enhances the capabilities of IoT systems by enabling efficient data processing, accurate pattern recognition, and adaptability to changing conditions. These techniques contribute to making IoT applications more intelligent, responsive, and resource-efficient. An autoencoder is a type of deep neural network that can be used to learn efficient data encoding in an unsupervised manner.

In this paper, two trained autoencoder models are compared in terms of their data reduction capabilities and their impact on machine learning tasks within the cloud server. Additionally, a comparative analysis is conducted between autoencoder models and principal component analysis (PCA) to explore variations between the two approaches.

Four primary scenarios are taken into consideration. The initial scenario represents the baseline, where dimensionality reduction is not applied. This scenario will be used to evaluate the other scenarios and compare our approach against the results of this baseline scenario. In the second and third scenarios, two different models of autoencoders are employed to reduce the image dimensionality on the edge server, and a machine learning (ML) task is run on the decoded images in the cloud. In the fourth scenario, principal component analysis (PCA) is used on the edge to encode images similar to the second and third scenarios. Then the cloud machine learning task is carried out on the encoded images. The scenarios are carried out for the task of object detection using a set of 4000 images randomly chosen from three different data sets. Results show that autoencoders can decrease network bandwidth without significantly affecting the accuracy of machine-learning tasks.

The rest of this article is organized as follows: “Background” provides background concepts, and “Related works” reviews related work. The methodology is described in “Methodology”, and the experiment and results are presented in “Experiment and results”. Finally, “Conclusion and future work” presents a conclusion and future work.

## Background

This section introduces some concepts about deep learning, principal component analysis, and edge-cloud architecture:

### Deep learning

Deep learning (DL) is highly valuable for learning complex models due to its ability to automatically extract complex patterns and features from data. Using neural networks with multiple layers, deep learning can discern hierarchical relationships within information, enabling the modeling of complex structures. This makes it particularly effective in tasks like image recognition, natural language processing, and pattern recognition, where understanding complex details is crucial^5^. The capacity of deep learning to learn from vast datasets and capture precise patterns allows it to play a vital role in various sectors, including healthcare, transportation, and others^6^.

The autoencoder (AE)^7^ is a valuable model in dimensionality reduction, simplifying complex datasets by capturing essential features. By learning efficient representations, autoencoders compress high-dimensional data into a lower-dimensional space. This reduction not only aids in preserving critical information but also accelerates computational processes. Autoencoders find applications in diverse fields, from image and signal processing to feature extraction, contributing to improved efficiency and streamlined analysis in various tasks.

The autoencoder takes the data, propagates it through a number of hidden layers to understand and condense its structure, and finally generates that data again. The autoencoder uses two types of networks: the first is called an encoder, and the other is a decoder, with the layers inside the encoder reflected in the decoder.

### Principal component analysis

Utilizing principal component analysis (PCA)^8^ in dimensionality reduction is a fundamental approach to streamline complex datasets and enhance computational efficiency. PCA identifies the principal components, which are linear combinations of the original features capturing the maximum variance in the data. By focusing on these key components, PCA allows for the reduction of data dimensions while preserving essential information. This process not only accelerates computational tasks but also aids in mitigating issues associated with high-dimensional data, such as the curse of dimensionality. In various applications, ranging from image and signal processing to machine learning, PCA proves instrumental in simplifying data representations, facilitating more effective analysis, and improving the overall performance of algorithms.

### Edge-cloud architecture

Edge devices^9^ play a pivotal role in the Internet of Things (IoT) ecosystem by bringing computational power closer to the data source. Unlike traditional cloud-centric models, edge computing allows for real-time processing and analysis of data at or near the point of origin. This minimizes latency, reduces the strain on communication networks, and enhances overall system efficiency. Edge devices enable quicker decision-making for applications like smart cities, healthcare, and industrial automation. By distributing computing tasks between the edge and the cloud, these devices contribute to a more responsive, scalable, and resilient IoT infrastructure. In line with this strategy, the edge device will be used to apply dimensionality reduction methods to the image data before sending it to the cloud.

## Related works

In the past few years, edge computing has been gaining considerable attention from both the research and industry sectors because it promises to reduce network traffic and latencies and reduce reliance on the cloud^10,11^.

Ghosh, Ananda, et al.^12^ proposed combining the edge-cloud architecture for IoT data analytics by leveraging edge nodes to reduce data transfer. To process data near the source, sensors are grouped according to locations, and feature learning is performed on the nearby edge node. They conducted experiments on a machine-learning task, specifically classification. The evaluation was performed on a task of human activity recognition from sensor data using the Mobile Health text-based dataset. The results demonstrated that the approach could reduce both data and the corresponding network traffic by up to approximately 80% with no significant loss of accuracy, especially when applying a large sliding window in the preprocessing phase.

Couturier, Salman, et al.^13^ implemented a denoising super-resolution deep learning model to restore high-quality images, with the application server receiving degraded images at a high compression ratio from the sender side. The experimental analysis demonstrates the effectiveness of this solution in enhancing the visual quality of compressed and downscaled images. As a result, the proposed approach effectively reduces the overall communication overhead and power consumption of constrained Multimedia Internet of Things (MIoT) devices.

Sood et al.^14^ propose a two-stage network traffic anomaly detection system compatible with the ETSI-NFV standard 5G architecture. Their architecture involves reducing dimensionality to compress the sample size at the edge of 5G networks, along with a deep neural network (DNN) classifier for detecting traffic anomalies. They utilized the UNSW-NB15 dataset and demonstrated that, with a dimensionality reduction factor of 81%, the achieved detection accuracy is 98%.

Sujitha, Ben, et al.^15^ proposed a method comprising two convolutional neural networks (CNNs) and a Lempel–Ziv Markov chain algorithm (LZMA)-based image codec. They presented a new image compression method for remote sensing using CNN. To balance image quality and compression efficiency, they used two CNNs, one on the encoding side and the other on the decoding side. The results proved the effectiveness of the presented method, which achieves an average peak signal-to-noise ratio (PSNR) of 49.90 dB and an average space-saving (SS) of 89.38%.

Krishnaraj et al.^16^ utilized a discrete wavelet transform (DWT)-based deep learning model for image compression on the Internet of Underwater Things (IOUT), achieving effective compression with better reconstructed image quality. A convolutional neural network (CNN) is utilized on both the encoding and decoding sides. The DWT-CNN model attains an average peak signal-to-noise ratio (PSNR) of 53.961 with an average space-saving (SS) of 79.7038%.

Zebang Song et al.^17^ demonstrated a lossy image compression architecture that leverages existing deep learning methods to achieve high coding efficiency. They designed a densely connected autoencoder structure for lossy image compression. Experiments show that their method significantly outperforms JPEG and JPEG2000 and can produce better visual results with sharp edges, rich textures, and fewer artifacts.

Fournier and Aloise^18^ proposed an empirical comparison between autoencoders and traditional dimensionality reduction methods. They evaluated the performance of PCA compared to Isomap and a deep autoencoder. For the evaluations, a K-Nearest Neighbor (KNN) classifier was used, and the results show that PCA computation time is faster than that of its neural network counterparts.

Some of the discussed studies did not use edge-cloud architecture integration with the IoT, and some of them focused on other data reduction methods without employing autoencoders or PCA. Additionally, some of them didn’t apply their evaluations to images. In contrast, our work explores the use of deep learning approaches for image dimensionality reduction on edge servers to decrease network traffic and latencies caused by data transfer to the cloud. We also apply an object detection machine learning task on the cloud to evaluate the approach.

## Methodology

This section introduces the methodology of the edge-cloud architecture and also presents methods for data reduction with the autoencoder and PCA.

**Figure 1:**
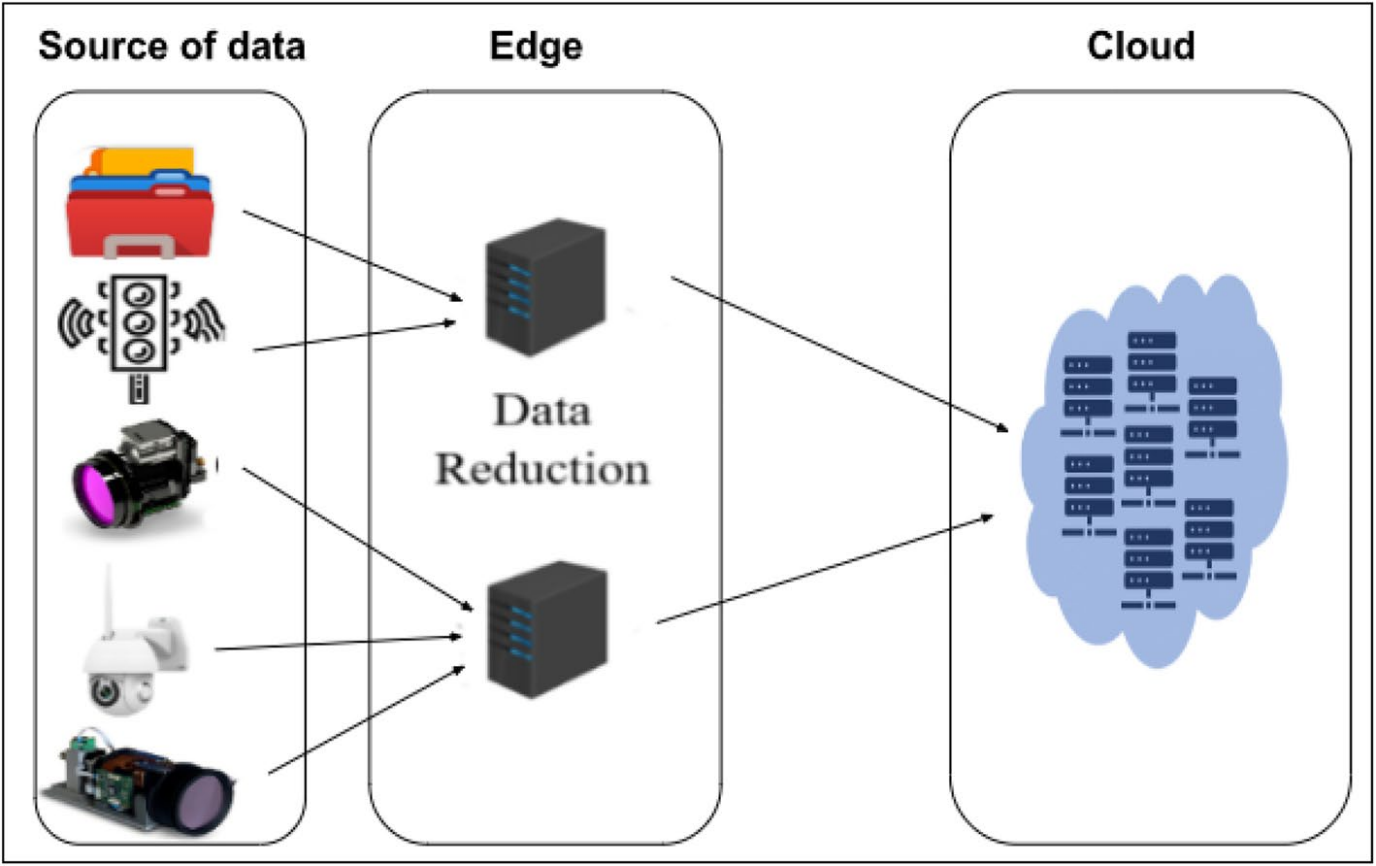
Edge-cloud architecture for IoT.

The overall architecture of the edge cloud is described in Fig. 1, illustrating its three main components: IoT sensors, serving as the data source; edge servers; and the cloud server. The initiation of the edge-cloud architecture involves receiving data from IoT sensors at the edge. The diagram further illustrates a potential scenario where data from diverse sensors is directed to various edge nodes, and all nodes subsequently forward this data to a centralized location. This setup allows machine learning tasks running in the cloud to benefit from data originating from various sources, including edge nodes. While specific tasks can be performed on the edge nodes, they would only have access to data from a subset of sensors. Data reduction can also occur at the edge to minimize the amount of data transmitted to the cloud.

**Figure 2:**
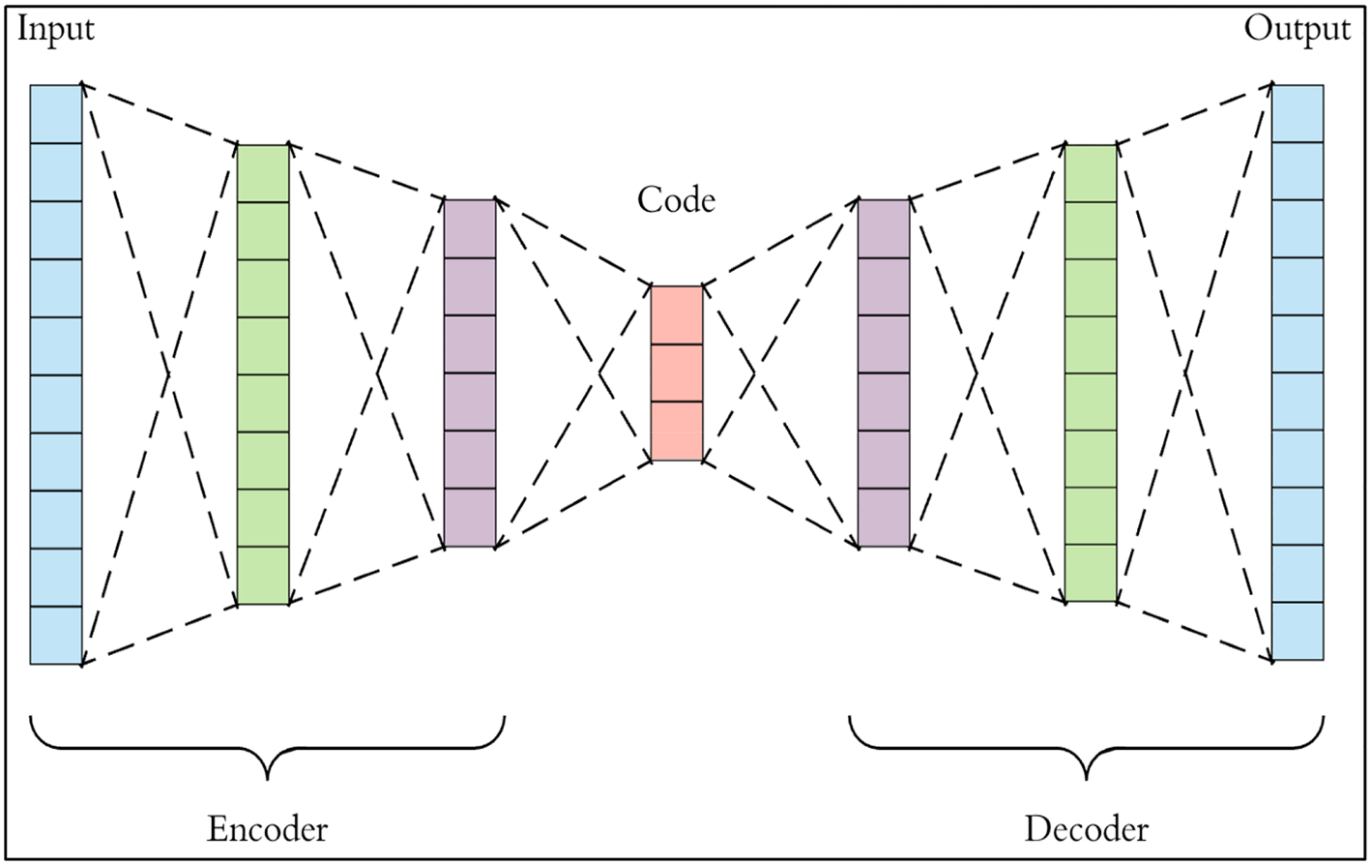
Autoencoder architecture.

Previous research has demonstrated the effectiveness of autoencoders and PCA in the field of dimensionality reduction. In some cases, autoencoders not only reduce dimensionality but can also detect repetitive structures^19,20^. Figure 2 describes the autoencoder architecture, which takes input data and processes it through several hidden layers. The number of neurons in the hidden layers is smaller than the number of neurons in the input layer, forcing an autoencoder to learn the internal structure of the data.

To integrate the autoencoder into the edge-cloud architecture, the encoder component of the network is located on the edge, while the decoder component is on the cloud. This way, when high-dimensional data arrives at the edge node, it is reduced to a smaller number of dimensions according to the encoder architecture. After this data is sent to the cloud, it can be reconstructed using the cloud-based decoder component of the autoencoder and then utilized for ML tasks.

A pre-trained model of the autoencoder was used in the experiments. Because autoencoder training requires a significant amount of time and computation, it must take place on high-spec devices such as the cloud or computers equipped with GPUs.

Principal Component Analysis (PCA) is a widely used linear dimensionality reduction technique. It is quicker and less expensive to compute than autoencoders. Also, it is quite similar to a single-layered autoencoder with a linear activation function.

**Figure 3:**
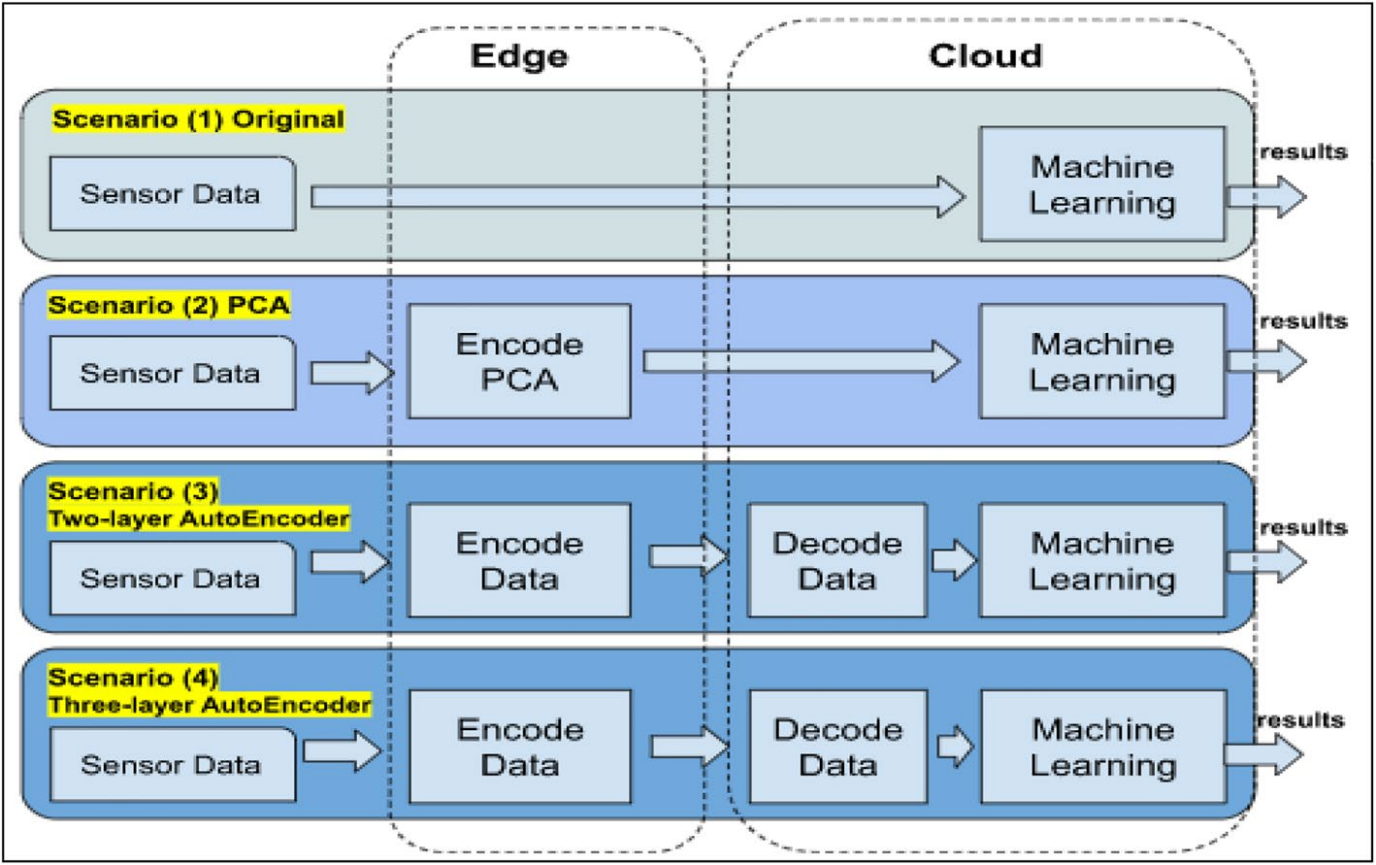
Computation models.

This paper explores four fundamental scenarios, as illustrated in Fig. 3.

### Scenario 1

This represents the default scenario, where image data from sensors is transmitted directly to the cloud server, and machine learning tasks are executed directly using the original data.

### Scenario 2

Data from sensors is sent to edge nodes, where data reduction is performed using principal component analysis (PCA). Encoded data is then sent to the cloud, where machine learning tasks are carried out with the encoded data.

### Scenario 3

The edge nodes perform dimensionality reduction on the data using a two-layer autoencoder, which is a trained model with two hidden layers. Machine learning tasks are then carried out on the cloud directly with the decoded data.

### Scenario 4

Similar to scenario 3, but utilizing a three-layer autoencoder at the edge. The three-layer autoencoder is a trained model with three hidden layers.

## Experiment and results

This section describes the experiments and the results.

### Experiment preparation

In our approach, a dataset comprising 6000 images has been used to train the autoencoder. The trained model will be used in the experiments to perform dimensionality reduction with the image data at the edge. The training performed on a machine has the following benefits:

- Nvidia GM107M [GeForce GTX 960 M]
- Intel CoreTM i7-6700HQ CPU @ 2.60 GHz
- 16 GB of RAM

And the training model parameters include:

- Optimizer: Adam
- Epochs: 50
- Activation: ReLu

The dataset, which comprises 6000 images, was selected from both the COCO and DIV2K datasets:

- The Microsoft Common Objects in Context (MS COCO) dataset^21^ is a large-scale dataset used for object detection, segmentation, key-point detection, and captioning. It comprises over 328K images with varying sizes and resolutions, each annotated with 80 object categories and five captions describing the scene.
- The DIV2K dataset^22^ comprises 1000 diverse 2K-resolution RGB images. All images were manually collected and have a resolution of 2K pixels on at least one axis (vertical or horizontal). DIV2K encompasses a wide diversity of content, ranging from people, handmade objects, and environments to natural scenery, including underwater scenes.

**Figure 4:**
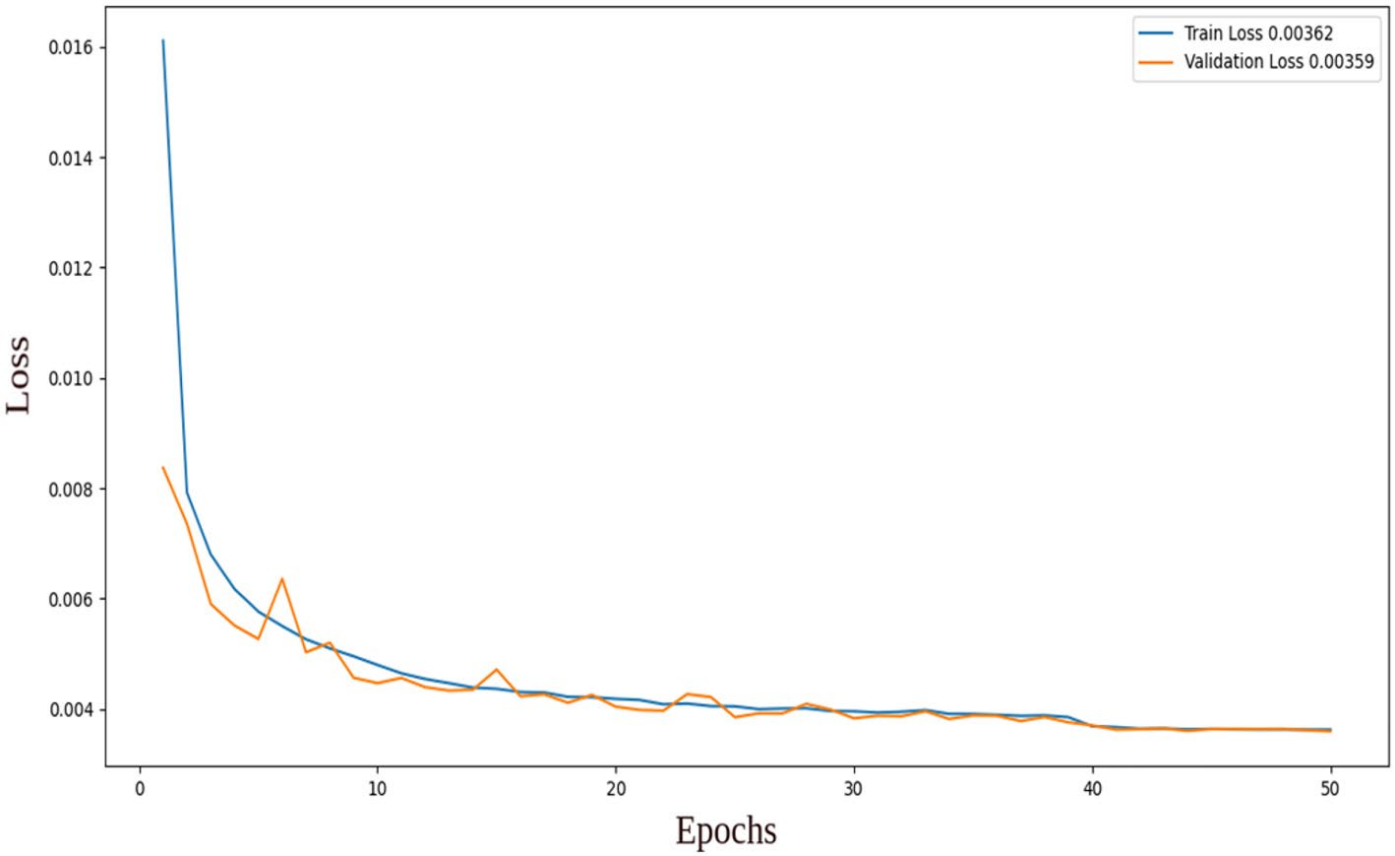
Training and validation loss for the two-layer autoencoder.

**Figure 5:**
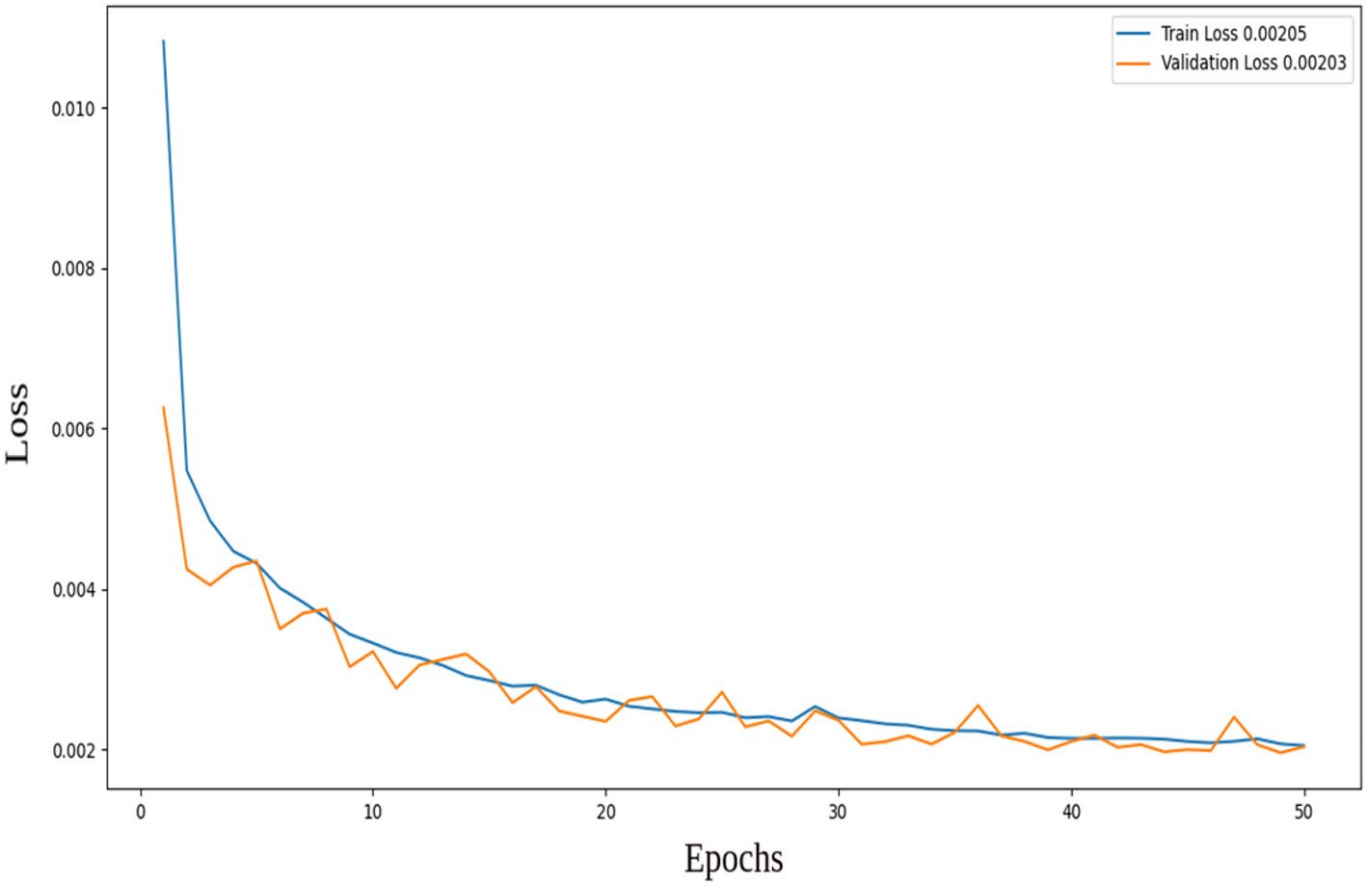
Training and validation loss for the three-layer autoencoder.

Figures 4 and 5 display the training and validation losses for the two-layer and three-layer autoencoders, respectively. In the two-layer autoencoder, the training loss was 0.00362, and the validation loss was 0.00359. For the three-layer autoencoder, the training loss was 0.00205, and the validation loss was 0.00203. Additionally, the Structural Similarity Index Measure (SSIM)^23^ is calculated for both models. SSIM is a method for predicting the perceived quality of digital television, cinematic pictures, and other types of digital images and videos. It is employed to measure the similarity between two images. The training shows that the Multi-Scale Structural Similarity Index Measure (MS-SSIM) on validation is 0.85716 for the two-layer autoencoder and 0.88425 for the three-layer autoencoder. This indicates a higher-quality reconstruction for the three-layer autoencoder compared to the two-layer autoencoder.

When we increased the number of epochs to more than 50 and the number of hidden layers to more than three layers, overfitting occurred. Increasing the epoch size and the number of hidden layers provides the model with more time to converge to an optimal solution, potentially resulting in improved accuracy. However, there is a risk of overfitting during training, where the model may become too specialized for the training data, capturing noise. This could lead to a reduction in accuracy on the validation or test set.

In machine learning, it is important to maintain the accuracy of the final machine learning task as high as possible. Since the primary objective of the proposed architecture is to reduce network traffic and latencies, considering the amount of data that can be reduced at the edge is also important.

### Machine learning task

The proposed approach has been evaluated for the task of image object detection using YOLO, which stands for ‘You Only Look Once’. YOLO is a technique employed for real-time object recognition and detection in various images. It treats object detection as a regression problem, providing class probabilities for observed images. Convolutional neural networks (CNN) are utilized in the algorithm for rapid object identification. As the name implies, the approach requires only one forward propagation through a neural network to detect objects^24^.

### Data sets

A set of 4000 images was used in the object detection task experiments, randomly chosen from three different datasets. The three datasets were selected to represent the diversity of the data used in the experiments. The datasets are:

- The MS COCO dataset^21^.
- The human detection dataset^25^ comprises 921 images from closed-circuit television (CCTV) footage, encompassing both indoor and outdoor scenes with varying sizes and resolutions. Among these, 559 images feature humans, while the remaining 362 do not. The dataset is sourced from CCTV footage on YouTube and the Open Indoor Images dataset.
- The HDA Person Dataset^26^ is a multi-camera, high-resolution image sequence dataset designed for research in high-definition surveillance. 80 cameras, including VGA, HD, and Full HD resolutions, were recorded simultaneously for 30 min in a typical indoor office scenario during a busy hour, involving more than 80 people. Most of the image data is captured by traditional cameras with a resolution of 640 × 480.

### Experiments

**Figure 6:**
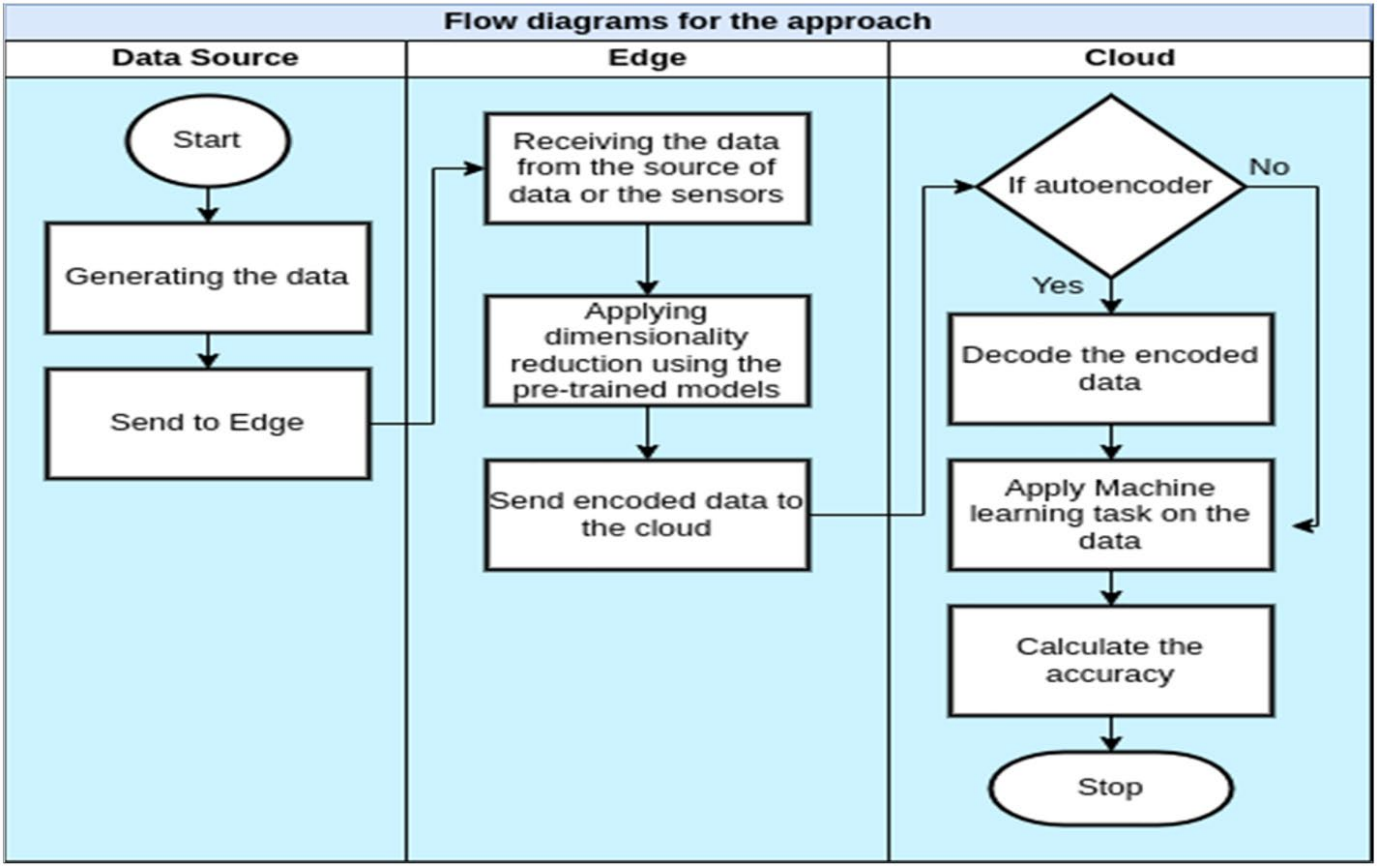
Flowchart diagram of the experiment.

**Figure 7:**
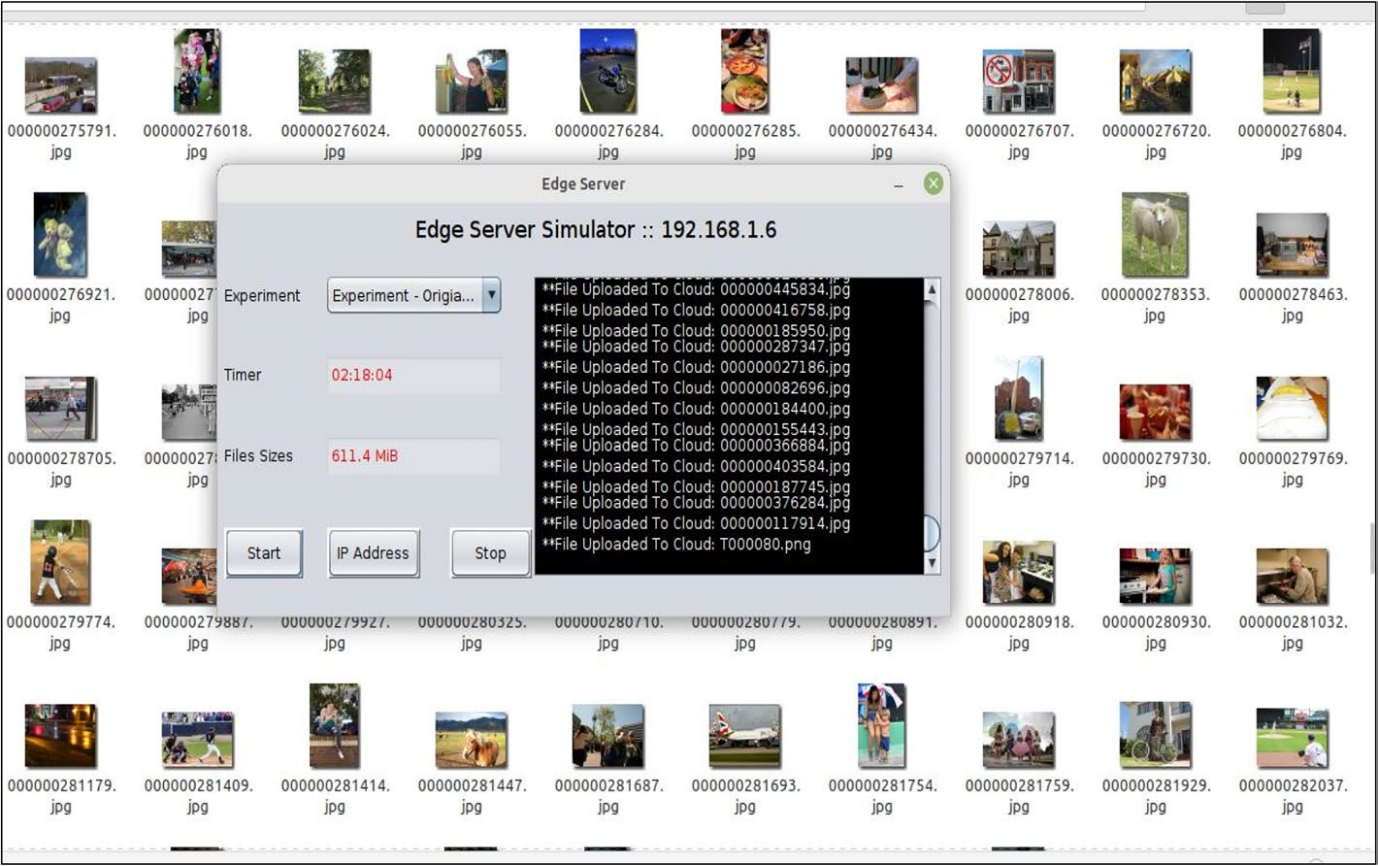
Simulations of edge device application.

The following four experiments were conducted, aligning with the four scenarios outlined in Fig. 3. The experiment was executed according to the flow in Fig. 6, starting with the data from the camera sensors or the existing collection of images. An Android mobile application was developed to run on Lenovo tablets, responsible for transferring images to the edge servers (via the edge node’s IP address and socket programming). Furthermore, the edge performs dimensionality reduction methods on the received images. Figure 7 shows a developed simulation desktop application used in edge nodes to receive images from sensors, manage the dimensionality reduction method, and transmit encoded data to the cloud server.

#### Experiment 1

Images are sent directly to the cloud from sensors, where an object detection task is performed on the data and the accuracy is measured. It will be used later to evaluate other experiments.

#### Experiment 2

The principal component analysis (PCA) is utilised at the edge nodes to instantly reduce the dimensionality of data, and then the encoded data is sent to the cloud. The object detection task was carried out on the encoded data in the cloud, and the accuracy was computed.

#### Experiment 3

Utilizing the encoder component of the autoencoder on the edge, the two-layer autoencoder encodes images in real time. The edge application directly transmits the encoded images to the cloud. Subsequently, the autoencoder’s decoder component operates in the cloud to decode the data. The decoded images are then used for the object detection task, and the accuracy is computed.

#### Experiment 4

Similar to Experiment 3, but employing the three-layer autoencoder.

The encoding and decoding times are taken into consideration for the autoencoder and PCA; the following three (Tables 1, 2, 3) provide examples of the encoding and decoding times for three different groups of images with different sizes and resolutions. It was noticed that the three-layer autoencoder’s encoding and decoding time was greater than the two-layer autoencoder’s in the chosen samples of images because it kept the quality of the decoded images close to the original ones.

**Table 1:** Group 1 (high resolutions).

	Encoding and decoding time for 10 images totalling 52.7 MB in size	
	Method	Time (seconds)
1	Two-layer autoencoder encode	16.1521
2	Two-layer autoencoder decode	50.0732
3	Three-layer autoencoder encode	21.3842
4	Three-layer autoencoder decode	62.3097
5	PCA encode	24.0819

**Table 2:** Group 2 (medium resolutions).

	Encoding and decoding time for 10 images totalling 2.6 MB	
	Method	Time (seconds)
1	Two-layer autoencoder encode	1.2671
2	Two-layer autoencoder decode	7.1150
3	Three-layer autoencoder encode	3.0814
4	Three-layer autoencoder decode	9.2671
5	PCA encode	2.1552

**Table 3:** Group 3 (low resolutions).

	Encoding and decoding time for 10 images totalling 107.1 KB	
	Method	Time (seconds)
1	Two-layer autoencoder encode	0.4440
2	Two-layer autoencoder decode	0.7247
3	Three-layer autoencoder encode	1.2618
4	Three-layer autoencoder decode	2.2730
5	PCA encode	0.5266

## Results

Two aspects of the system were evaluated: the impact of data reduction on the ML task accuracy and the degree of data reduction. For experiment 1, the accuracy, recall, precision, and F1-Score were calculated, and the results were all the same, or very close, at 93.06%. For experiment 2, the results were 84.66%. For experiment 3, the results were 87.63%. And for Experiment 4, the results were 89.14%.

**Figure 8:**
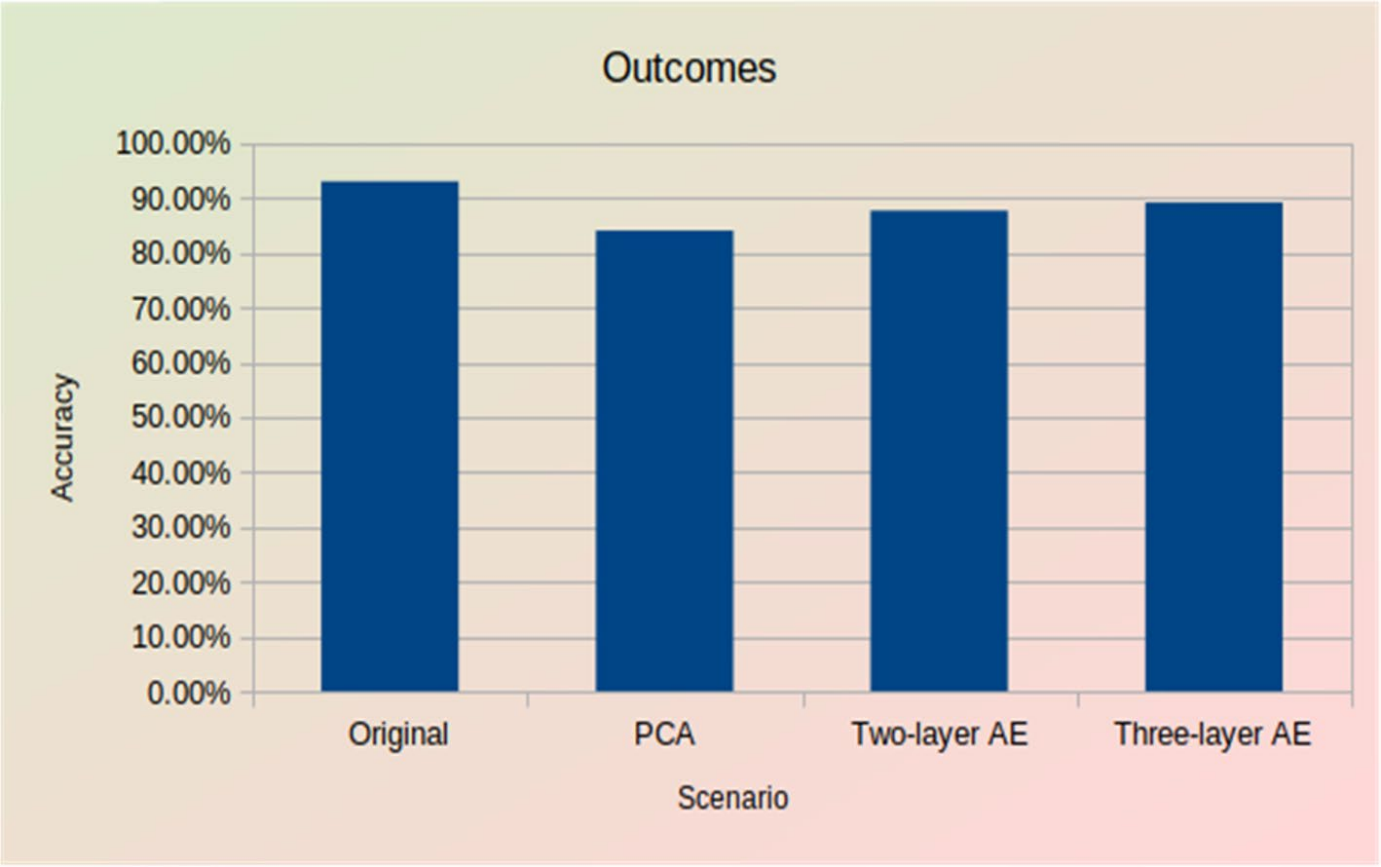
Accuracy outcomes.

The accuracy of the object detection task using an autoencoder and PCA is compared in Fig. 8. It is noticeable that when using the three-layer model of the autoencoder, the machine learning task achieved good accuracy close to the original scenario, which is better than using the two-layer model. However, when using PCA to encode the data, the machine learning task achieved less accuracy than an autoencoder.

As the results show, increasing the number of hidden layers in the autoencoder model by one improves the quality of the autoencoder’s latent representation when decoding the data and results in high machine-learning accuracy.

**Figure 9:**
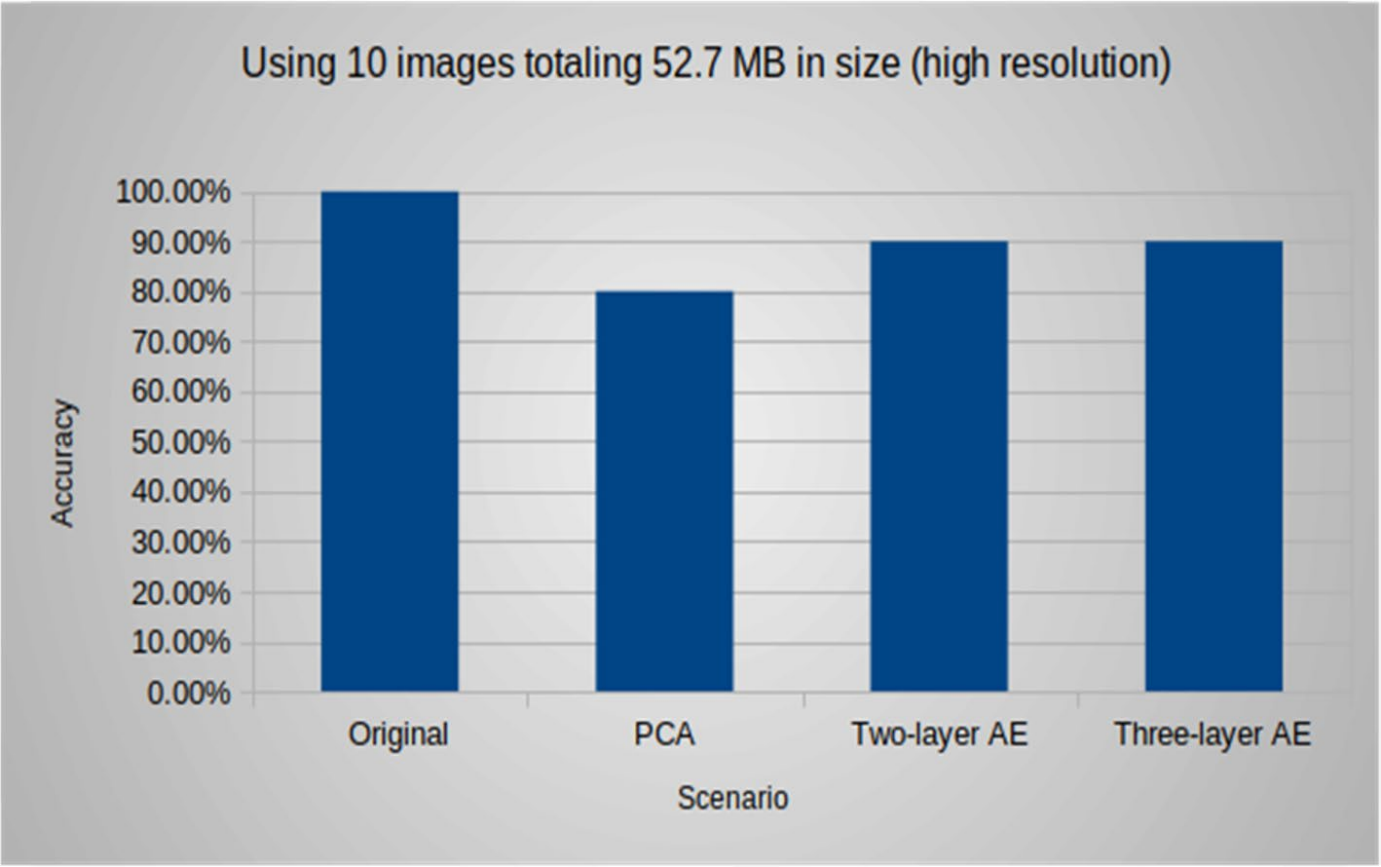
Group #1: accuracy of the object detection task using 10 images totaling 52.7 MB in size (high resolution).

**Figure 10:**
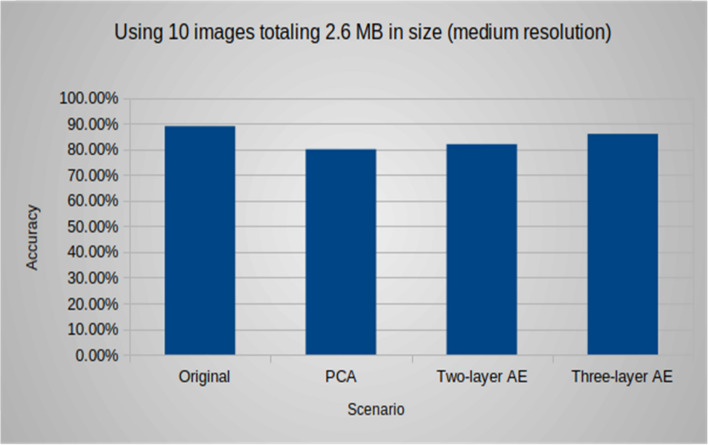
Group #2: accuracy of the object detection task using 10 images totaling 2.6 MB in size (medium resolution).

**Figure 11:**
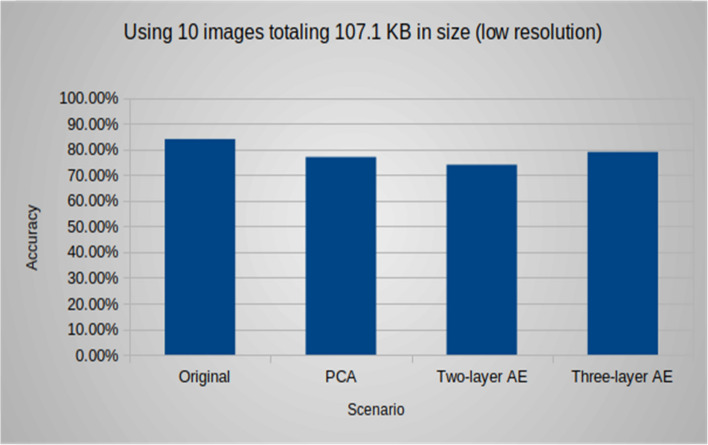
Group #3: accuracy of the object detection task using 10 images totaling 107.1 KB in size (low resolution).

Additionally, the following (Figs. 9, 10, 11) compare the accuracy of the object detection task using different groups of images with various sizes and resolutions. In group #1, the accuracy for the native experiment was 100, while using both models of the autoencoder resulted in an accuracy of 90, and it was 80 when using PCA. For group #2, the accuracy for the native experiment was 89, and when using the two-layer and three-layer autoencoders, the accuracy was 82 and 86, respectively, and it was 80 when using PCA. In group #3, the accuracy for the native experiment was 84, and when using the two-layer and three-layer autoencoders, the accuracy was 74 and 79, respectively, and it was 77 when using PCA. It was observed that an increase in image resolution enhances the quality of the decoded images produced by the autoencoder decoder part, resulting in improved accuracy for object detection tasks.

**Figure 12:**
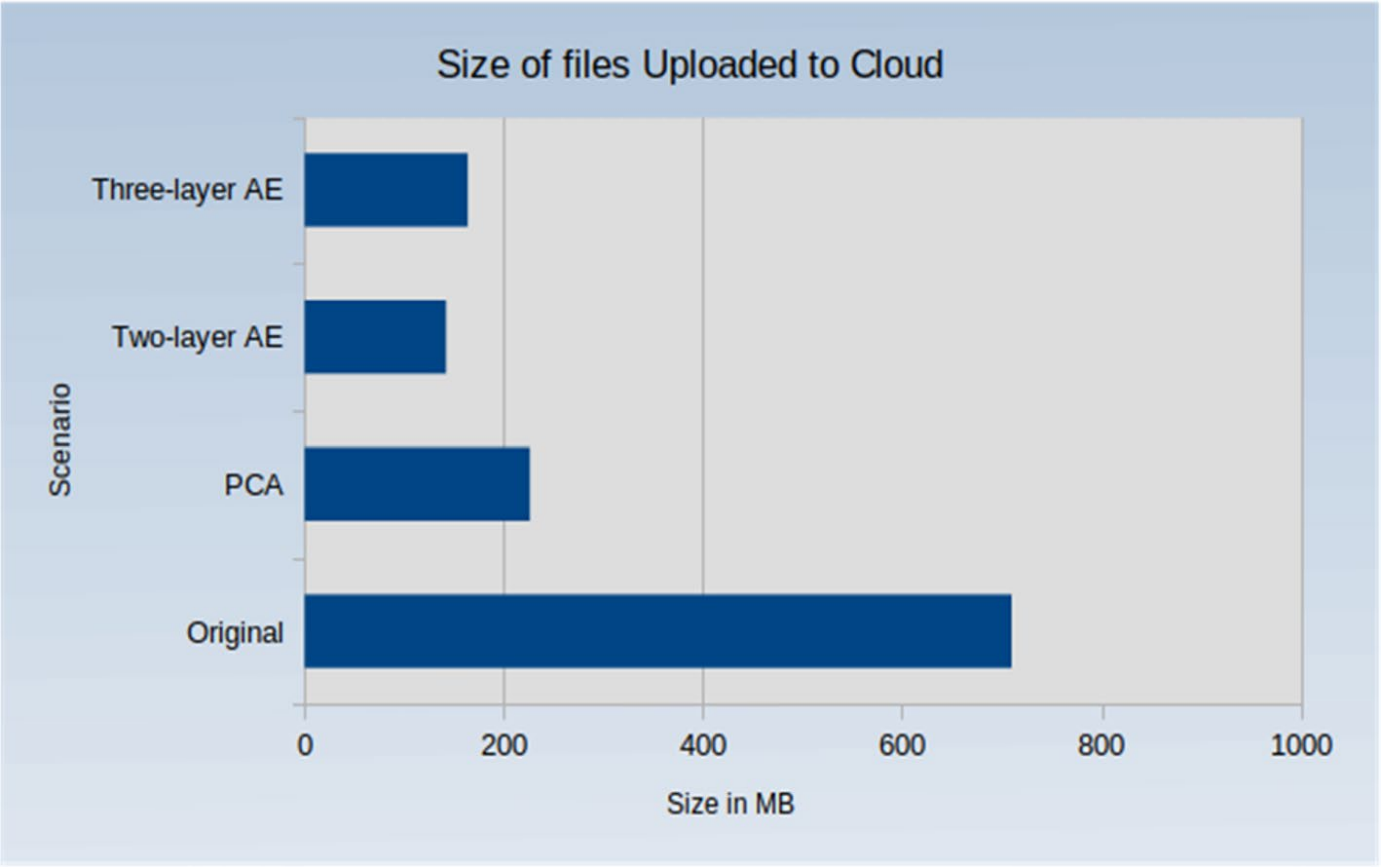
Data size for different scenarios.

**Figure 13:**
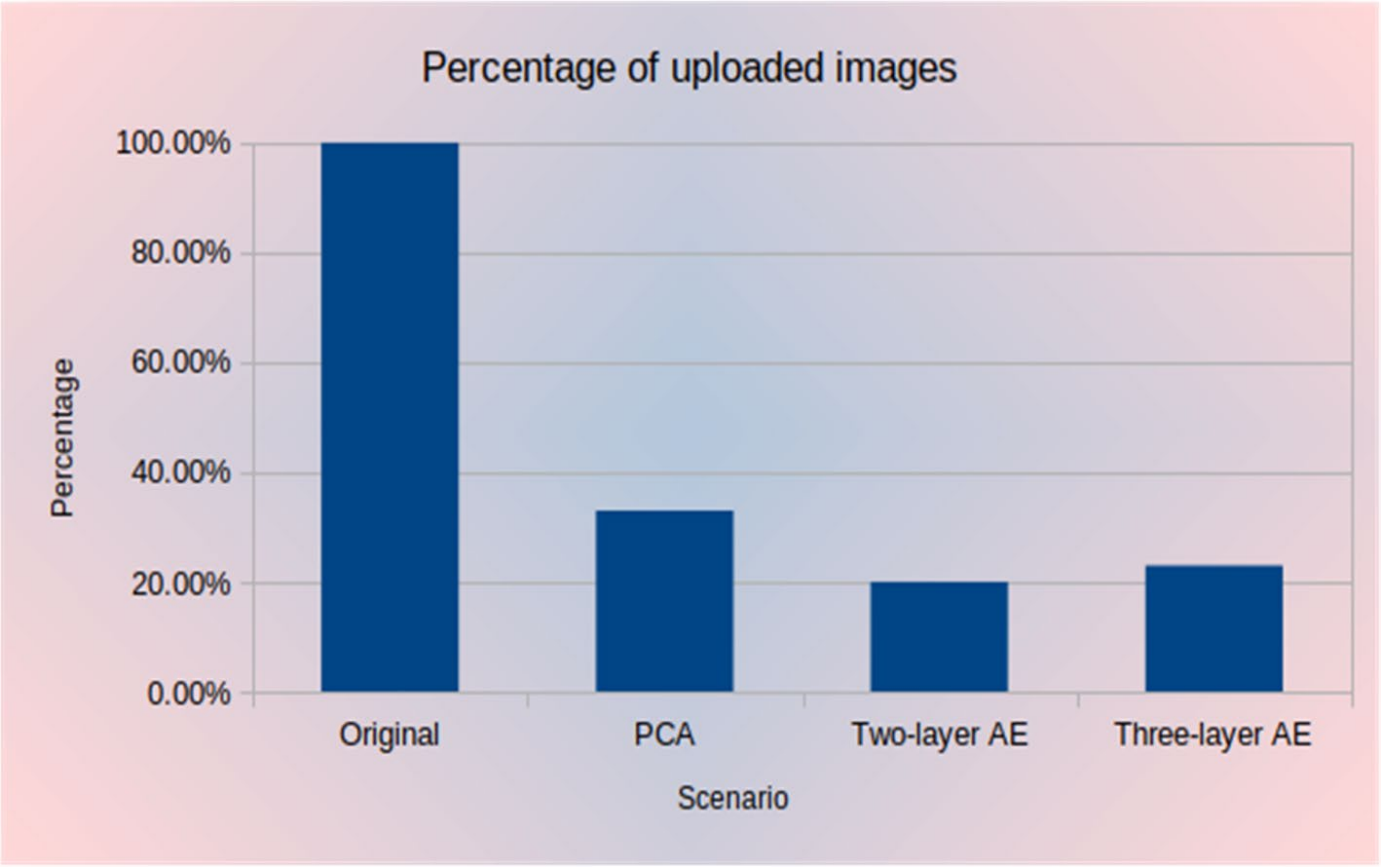
Percentage of uploaded data.

Because the main objective of our approach is to reduce network traffic and latencies, it is important to examine how much the proposed approach reduces data size. Figure 12 compares the uploaded original data size to the total size for other experiments. Figure 13 shows the percentage of the total size of uploaded images. It can be seen that the data is reduced from 710 MB for the original data to 142.1 MB when using the two-layer autoencoder (i.e. an 80% reduction), 163.9 MB when using the three-layer autoencoder (i.e. a 77% reduction), and 226.3 MB when using PCA (i.e. a 68% reduction). Consequently, the data sent to the cloud is significantly reduced, which is especially important in the case of large data quantities such as those in the IoT.

The experiments presented here show that, by using autoencoders, we were able to reduce the dimensionality of the images without significantly impacting the accuracy of machine-learning tasks. Additionally, images with high resolution and quality exhibited better results than images with low quality in terms of object detection tasks and the autoencoder decoder components when decoding the encoded data. Based on these outcomes, it is clear that applying this approach can effectively lower both the bandwidth usage and storage needs of IoT devices. Moreover, increasing the compression rate in a deep learning autoencoder for images will improve storage efficiency and faster transmission, but at the cost of decreased image quality and potential loss of information. The trade-off between compression and image fidelity needs to be carefully managed based on the goals and constraints of the particular application or use case.

## Conclusion and future work

Massive amounts of data have been generated through data collected across IoT applications, mostly through the sensors connected to the devices, and this trend is expected to continue. There will be an increase in network traffic and latency if all of this data is attempted to be transferred to the cloud for processing and storage.

To address these challenges, this work proposes combining edge and cloud architectures for IoT and utilizing machine learning, specifically autoencoders and PCA, to reduce the quantity of data sent to the cloud. The autoencoder’s encoder component is placed at the edge. Afterward, the data is transferred to the cloud for additional processing. The original data can be restored using the decoder component of the autoencoder and then used directly for the machine learning task, such as object detection. The proposed approach was evaluated on a set of 4000 images randomly chosen from three datasets: COCO, human detection, and HDA datasets.

Results show that the autoencoder model is capable of significantly reducing the size of uploaded images without a significant impact on machine learning task accuracy.

The suggested approach is used and examined only for images. Future research will explore how the suggested approach might be applied to various types of data. Moreover, the research will examine how the suggested methodology might be used for various machine learning tasks and with various datasets.

## Data Availability

The datasets used or analysed during the current study are available from the corresponding author upon reasonable request.
